# qs*GW* quasiparticle and *GW*-BSE excitation energies of 133,885 molecules

**DOI:** 10.1038/s41597-026-07018-4

**Published:** 2026-03-10

**Authors:** Dario Baum, Arno Förster, Lucas Visscher

**Affiliations:** Vrije Universiteit Amsterdam, Department of Chemistry and Pharmaceutical Sciences, De Boelelaan 1108, 1081 HZ Amsterdam, The Netherlands

## Abstract

Machine learning applications in the chemical sciences, especially when based on neural networks, critically depend on the availability of large quantities of high-quality data. As they provide excellent accuracy for both charged and neutral excitations, a large dataset containing quasiparticle self-consistent GW (qs*GW*) and Bethe-Salpeter equation (BSE) data would be highly desirable to model excited state energies and properties. In this work, we introduce a dataset for qs*GW*-BSE excitation energies and qs*GW* quasiparticle energies of unprecedented size. Our dataset, denoted QM9GWBSE, supplies *GW*-BSE singlet-singlet and singlet-triplet excitation energies, corresponding transition dipole moments and oscillator strengths as well as qs*GW* quasiparticle energies for all molecules from the popular QM9 dataset. We anticipate that QM9GWBSE will provide a solid foundation to train highly accurate machine learning models for the prediction of molecular excited state properties.

## Background & Summary

Accurate prediction and description of neutral and charged excitation energies is crucial for understanding light-matter interactions, charge transport, and spectral properties in molecular and condensed-phase systems. Computational methods enable the rational design of functional materials and provide insight into processes that are often inaccessible to direct experiment, for instance in photovoltaics^1^ or photosynthesis^2,3^. Rational design often requires searching for certain properties in large chemical spaces of hundreds of thousands of molecules. At the ab initio level, equation-of-motion (EOM) coupledcluster (CC)^4,5^ methods or its similarity-transformed variants (STEOM-CC)^6,7^ are considered the gold standard for the calculation of excited-state properties, since they converge to the full configuration interaction (FCI) limit with increasing orders of excitation rank^8–10^. Unfortunately, even truncated versions like EOM-CCSD (single and double excitations) EOM-CCSDT (single, double, and triple excitations) suffer from steep computational scaling of *N*^6^ and *N*^8^ respectively, with *N* denoting the system size, making their use in large-scale screening studies impossible. For this reason, time-dependent^11,12^ density-functional theory^13,14^ (TD-DFT) is often the method of choice for such workflows^15,16^, sometimes in combination with cheaper tight-binding based approaches^17,18^. However, while TD-DFT is a relatively cheap method from an ab initio perspective, it is still computationally expensive. On top of that, it is often not accurate enough^8^ and suffers from an undesirable dependence on the choice of density functional.

Motivated by such complications, data-driven methods for the application in quantum chemical simulations have recently gained considerable attention. In particular, neural network models offer the prospect of drastically reducing the cost of electronic-structure and excited-state simulations while maintaining a level of accuracy that is often comparable to the first-principles methods which have been used for their parametrization^19–24^. These approaches thus hold the promise to accelerate materials and molecular discovery without sacrificing predictive power. However, training accurate neural network models for such applications requires sufficiently large datasets of adequate data quality. Ideally, experimental data should be used for this purpose but unfortunately, experiments are usually much too costly and involved to produce sufficiently large datasets.

Therefore, one usually resorts to computational methods as surrogates for experimental references. TD-DFT provides an affordable alternative that could be used to generate databases of sufficient size^25,26^, but it is limited by its often insufficient accuracy. Unfortunately, producing datasets of adequate size for neural network training with highly accurate wave function-based methods is likewise unfeasible due to the aforementioned computational demand. The largest and most accurate dataset of neutral excitation energies of near FCI quality is the QUEST database, consisting of roughly 1500 excitation energies of small to medium molecules^8,9,27,28^, which is insufficient to train neural networks. Orders of magnitude larger is the GDB-9-Ex_EOMCCSD dataset^29^. It contains STEOM-CCSD excitation energies of  ~ 80 000 molecules of the GDB-9 database with automated active space selection^30^. STEOM-CCSD reaches errors of only 50-200 meV in typical benchmarks for different classes of excitation energies and is usually more accurate than EOM-CCSD, whose accuracy diminishes when going from small to medium molecules^31^. In search for an electronic structure method which can be used to calculate even larger databases of accurate excited state energies and properties, the *GW* approximation (GWA) to the electronic self-energy^32–35^ combined with the Bethe-Salpeter equation (BSE)^36–39^ provides an affordable middle-ground. The GWA gives access to molecular charged excitations like ionization potentials (IP) and electron affinities (EA), and the subsequent solution of a BSE with the *GW* quasiparticle (QP) energies and statically screened electron-hole interaction as input gives access to excited states and absorption spectra. Most *GW* calculations neglect the off-diagonal elements of the self-energy and calculate QP energies as a perturbative correction to the Kohn–Sham (KS) eigenvalues (*G*_0_*W*_0_)^40,41^, often combined with an iterative update of the *G*_0_*W*_0_ eigenvalues [eigenvalue self-consistent *GW* (ev*GW*)]^42^. Careful selection of the KS starting point, results in QP energies with deviations of only 100-200 meV compared to highly accurate reference values in weakly correlated systems^43–48^. Also for singlet-singlet neutral excitation energies, *GW*-BSE matches the accuracy of the more expensive STEOM/EOM-CCSD methods^46,49– 51^, or even outperforms them^52^ while it is less accurate for singlet-triplet excitations^49,50,53–55^. Despite this excellent trade-off of accuracy and computational efficiency, datasets of *GW*(-BSE) QP energies and excitation energies of sufficient size to train neural networks are rare. Examples of publicly available *GW* datasets are the GW5000 subset of the OE62 dataset^56^ and the dataset of Fediai *et al*.^57^. The GW5000 subset of the OE62 dataset provides *G*_0_*W*_0_@PBE0^58–60^ quasiparticle energies for 5000 molecules with up to 100 atoms. While being especially useful for benchmarking purposes^61^ or to train Δ-ML models^62,63^, such data quantity usually is not enough for training neural networks for end-to-end prediction, i.e. prediction of the desired property directly from the molecular structure^64–66^. Exceptional in this regard is the dataset by Fediai *et al*. which contains IPs and EAs for all of the 133 885 data points in the QM9 database^67,68^ calculated using ev*GW*@PBE. In a follow-up paper, they demonstrated their dataset to be sufficient for training robust and accurate DimeNet++ and SchNet models^69–72^. However, neutral excitation energies calculated with the BSE are missing as of yet.

To remedy this deficiency, we present here the largest *GW*-BSE dataset to date: we provide *GW*-BSE singlet-singlet and singlet-triplet excitation energies together with transition dipole moments and oscillator strengths for the complete QM9 dataset. Rather than using ev*GW*, we thereby decided to perform all of our *GW* calculation in a quasi-particle self-consistent fashion (qs*GW*) which self-consistently updates both QP energies and corresponding orbitals^73–76^. ev*GW* largely removes the dependence on the starting point for QP energies and frequently leads to excellent agreement with high-accuracy reference values^77–81^, while the effect of updating also the orbitals is usually rather small^82^. For neutral excitation energies, the situation is however a bit more nuanced. While for Thiel’s set^83^ the mean average deviation between ev*GW*@PBE-BSE and ev*GW*@PBE0-BSE neutral excitation energies was found to be as small as 80 meV^79^, more recent work^84,85^ found a much stronger dependence of *GW*-BSE excited state energies on the KS orbitals. Adopting the only slightly more expensive^86^ qs*GW* approach overcomes the dependence of QP and excited-state energies and properties on the choice of a density functional in diagonal approximations to the self-energy^43,44,53,87^. Even though performance for small molecules is mixed^88^, for a standard benchmark set of QP energies of 24 organic acceptor molecules (Acc24)^89^ representative for those present in the QM9 dataset, the qs*GW* method has been shown to be among the most accurate of all *GW* methods^48,90^. For this dataset, Ref. ^48^, reported a MAD of 90 meV, and Ref. ^90^ of 120 meV for this set, on par with *G*_0_*W*_0_ based on optimally-tuned range-separated hybrid approaches reported by different authors^43,47,91^. For a more detailed comparison also see Table 1 in Ref. ^90^. qs*GW* also performs very well for the GW100 set^92^, with MADs of 220 meV reported in Ref. ^44^, and of 160 meV reported in Ref. ^93^ using the related joint approximate diagonalization approach. Finally, we mention that the GW100 benchmarks find qs*GW* to overestimate IPs by about 60-100 meV on average^44,93^, while no such tendency has been observed for the Acc24 benchmarks^48,90^.

**Table 1:** Overview of properties in the QM9GWBSE dataset with corresponding units and filenames in the data repository.

Filename	Description	Unit
eqp	qs*GW* quasiparticle energies	eV
eexc_ss	qs*GW*-BSE singlet-singlet excitation energies	eV
eexc_st	qs*GW*-BSE singlet-triplet excitation energies	eV
trans_dip_mom	Transition dipole moment vectors of singlet-singlet excitations	D
osc_stren	Oscillator strengths of singlet-singlet excitations	—
edft	DFT MO energies	eV
xyz	Molecular structures	—

Moreover, qs*GW*-BSE provides highly reliable excited-state energies across a wide range of molecular systems^50,51,90^. Overall, qs*GW*(-BSE) constitutes a robust, parameter-free framework for predicting accurate quasiparticle and excitonic properties, with an excellent trade-off between accuracy and computational effort. For this reason, our dataset offers an unprecedented combination of high data quantity, quality, and diversity of properties, making it an excellent choice for developing neural-network-based models for a variety of applications in molecular spectroscopy.

## Methods

All calculations were performed with the ADF engine of the Amsterdam modeling suite (AMS)^94^ via the PLAMS toolkit^95^. We downloaded the molecular structures of the QM9 dataset^96^ from the official repository (10.6084/m9.figshare.c.978904.v5, specifically the dsgdb9nsd.xyz.tar.bz2 file). The QM9 molecular structures are optimized at the B3LYP/6-31G(2df,p) level of theory^68^. For the DFT step preceding the *GW* calculations, we used the BHandHLYP functional^97,98^ as implemented in libXC^99^. For the subsequent qs*GW* and qs*GW*-BSE steps, we used the respective implementations in ADF^51,61,86^. For both, we used the TZ3P basis set^100^. We used minimax grids with 16 points in imaginary time and imaginary frequency, and evaluated the self-energy on the real axis through analytical continuation with a 16-point Padé approximant^100^, following the algorithm by Vidberg and Serene^101^. For all qs*GW*-BSE calculations, we use the default value of 10 for the maximum number of iterations in the qs*GW* part and the direct inversion iterative subspace (DIIS) method^86,102,103^ for convergence acceleration. Further, the maximum number of iterations of the Davidson algorithm is set to 20. We calculate the lowest 5 excitation energies for both singlet-singlet and singlet-triplet excitations.

For other numerical settings, we distinguish two cases: initially, we run qs*GW*-BSE calculations for all molecules with the numerical quality set to *Good*, and eliminate almost linear dependent products of basis functions from the primary basis by setting a *K*-matrix regularisation parameter^104^ to *ϵ*_*K*_ = 5 × 10^−3^. This entails the use of an auxiliary basis consisting of auxiliary functions with angular momentum up to *l* = 4^105^ in the pair atomic density fitting (PADF)^104^ approximation to the 2-electron integrals on which our implementation is based^61^. We choose this setting as the default because it is very efficient and usually reliable^51,86^. However, the auxiliary basis should ideally contain auxiliary functions with angular momenta *l* = 5 and *l* = 6 to be able to accurately represent products involving the *f*-functions contained in the TZ3P basis set for second-row elements. The lack of these functions can hinder convergence of either the qs*GW* calculation or the Davidson diagonalization, or, in the worst case, induce a variational collapse. We detect those rare cases using a variety of automatic filters described in Section *Technical Validation*. All calculations that trigger one of our automatic filters are restarted with *ϵ*_*K*_ = 10^−3^, and the numerical quality set to *VeryGood*, entailing the use of an auxiliary basis with functions of with angular momentum up to *l* = 6^105^. With these settings, all qs*GW*-BSE calculations can be safely converged with numerical errors that are at least an order of magnitude smaller than the errors inherent in the method. For full reproducibility, we include the ADF input files for both the initial calculations and the restart calculations in the SI.

## Data Records

The dataset is publicly available at Zenodo (10.5281/zenodo.17902233)^106^. The repository contains a zip file for each property. Each zip file includes a data file for each molecule with the filenames being mol_ID where ID is the same numerical molecule identifier as in the QM9 dataset with leading zeros left out (e.g. 4200 instead of 004200). Each data file contains the numerical values of the property at hand as a 1D-array in ascending order, so from lowest to highest energy (e.g. lowest occupied to highest virtual orbital energy or lowest to highest excitation energy). Table 1 maps each zip file name to its respective property with the corresponding unit.

## Technical Validation

The large amount of calculations performed in this work prevents manual inspection of all generated data. Therefore, we ensured data quality by including automated filters to check the physical plausibility of each calculation as well as statistical analysis of the data, ensuring that no outliers or systematic artifacts are present, and to compare our work to the current state of the art.

### Automated Filters

We used several automated checks to detect and restart calculations with unphysical results. The checks are based on the physical plausibility of the quasiparticle energies of the *GW* calculations in relation to the MO energies of the underlying DFT calculations. First, for organic molecules, the gap between the qs*GW* highest occupied molecular orbital (HOMO) and lowest unoccupied molecular orbital (LUMO) is almost always larger than the ones of hybrid functionals like BHandHLYP with 50 % exact exchange^86^. Second, the *GW* HOMO QP will be lower than the corresponding DFT MO energy, and the LUMO energy will be higher^86^. Third, within the QM9 set of molecules, the HOMO QP energy should not be lower than  − 20 eV to be plausible. With these simple checks, we can detect all cases of variational collapse due to an insufficient auxiliary basis set or of convergence of the qs*GW* calculation to an unphysical solution^86^. Additionally, we checked for imaginary eigenvalues in the Davidson algorithm, which would certainly arise from a variational collapse in the qs*GW* calculation.

Any calculation that triggered one or more of these filters (less than a percent) was restarted with the tight settings described in the *Methods* section. For such restarted calculations, the exact same quality checks were applied. In all but two cases, restarting with tight settings led to a normal termination of the calculation, passing all filters. For both outliers, mol_37992 and mol_133858, only the *GW*-BSE part of the calculation failed due to imaginary eigenvalues in the Davidson procedure. The prior qs*GW* calculations converged as expected with reliable results in both cases. The outliers have a qs*GW* QP HOMO-LUMO gap of 4.532 eV and 5.847 eV, respectively, which is substantially lower than the mean of 11.146 eV over the whole dataset. Additionally, both molecules display negative QP LUMO energies. We obtain equivalent results, low QP gaps with negative LUMOs, for equivalent *G*_0_*W*_0_ calculations. Furthermore, TD-DFT calculations for both molecules also fail for the singlet-triplet calculations also with imaginary eigenvalues in the Davidson algorithm. All in all, this indicates the existence of a singlet-triplet instability. The two outlier cases are specified in the README file of the data repository.

### Basis Set Convergence

**Fig. 1:**
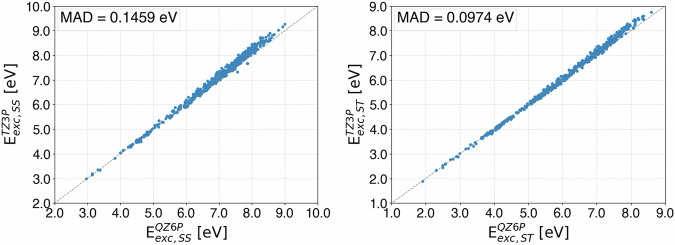
Deviation of *GW*-BSE singlet-singlet (left) and singlet-triplet (right) excitation energies based on the TZ3P and QZ6P basis sets.

To validate the choice of the TZ3P basis set for *GW*-BSE calculations, we demonstrate that it is close to convergence with respect to the basis set quality. For this purpose, we randomly sample 500 QM9 molecules and perform *GW*-BSE calculations with the exact same settings as for our dataset but with the QZ6P basis set instead of the TZ3P basis set and with the numerical quality set to *VeryGood* instead of Good. Generally, *GW*-BSE excitation energies with QZ6P can be considered converged in terms of basis size and are therefore a suitable yet feasible target for this quality check^51^. We thus compare all resulting singlet-singlet and singlet-triplet test excitation energies with the respective TZ3P equivalents from our dataset. The results are plotted in Fig. 1. A quantitative breakdown of the mean relative errors in different excitation energy ranges is provided in Tables S1 and S2 in the Supporting Information. Especially for lower excitation energies, we get excellent agreement between QZ6P and TZ3P for both singlet-singlet and singlet-triplet excitations. For higher-lying excitation energies, the deviations grows a bit larger in both cases but are still fairly small. The average deviation is of the same order of magnitude as the typical errors of accurate *GW*-BSE calculations with respect to highly accurate wave function-based reference values^46,50,90,107^. Higher excitation energies often correspond to excitations to diffuse virtual orbitals and might have substantial Rydberg character. In such cases, more diffuse basis functions are needed to relax the corresponding orbitals. That explains why, especially for these cases, in the TZ3P basis, the excitation energies are slightly overestimated compared to the results using the QZ6P. We also note that, as all *GW*-BSE methods, also qs*GW* tends to underestimate singlet-triplet excitations^50,53^. For this reason, the basis set incompleteness error in our calculations results in a favourable error cancellation. For singlet-singlet excitations, qs*GW*-BSE does not exhibit any clear trend to either over- or underestimate excitation energies.

### Data Analysis

We assess the overall consistency of the data by analyzing distributions of properties to confirm the absence of outliers and systematic errors. Furthermore, we compare those contributions to equivalent state-of-the-art work to demonstrate the plausibility of our results. For comparison of the QP energies, we choose the work by Fediai *et al*.^57^ who also computed *GW* QP energies for the QM9 set of molecules, but using *G*_0_*W*_0_@PBE and ev*GW*@PBE instead of qs*GW* as in our case. They extrapolated the resulting *GW* QP energies to the complete basis set (CBS) limit using a commonly used two-point extrapolation scheme that presupposes dependence of the basis set incompleteness error on the inverse of number of basis functions^48,56,92,100,108–113^. They performed the extrapolation using the aug-cc-DZVP and aug-cc-TZVP basis sets, which are usually too small to allow for a reliable extrapolation^114^. This is also apparent from the calculations by Fediai *et al*., who point out severe outliers in their CBS extrapolation of both *G*_0_*W*_0_@PBE and ev*GW*@PBE QP HOMO and LUMO energies^57^.

**Fig. 2:**
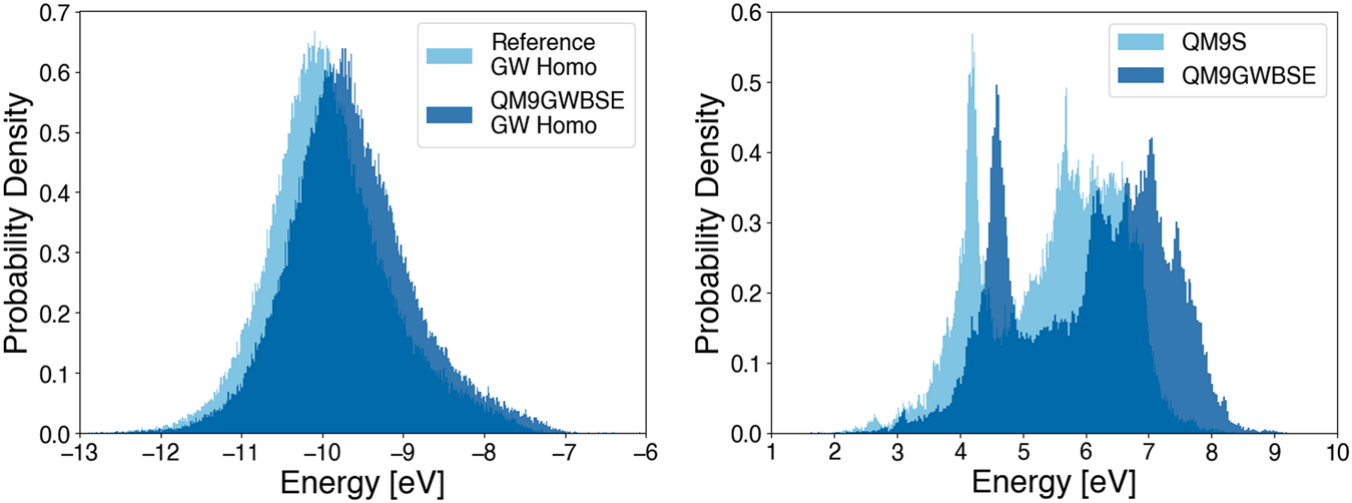
Distribution of GW HOMO energies from the QM9GWBSE and the chosen reference dataset (left) and distribution of *GW*-BSE excitation energies from the QM9GWBSE dataset and TD-DFT excitation energies from the QM9S dataset (right).

On top of that, recent work has demonstrated that for qs*GW*, the basis set convergence of QP energies heavily depends on the molecule at hand^114^. Therefore, the two-point extrapolation^115,116^ cannot be applied without introducing a considerable amount of outliers, which would result in unreliable data quality. This would even be the case if a QZ basis set had been used in the extrapolation^114^. Also the recently introduced data-driven extrapolation schemes, which use the orbital kinetic energy as a descriptor for basis set incompleteness^114,117^ do not work well for qs*GW*^114^. For this reason, we did not extrapolate our QP energies to the CBS limit. Figure 2 on the left shows the distribution of ev*GW*@PBE HOMO QP energies with CBS extrapolation from Fediai *et al*. and the qs*GW* QP HOMO energies from our work. As can be seen, both their and our datasets agree qualitatively on the form of the distributions. On average, the qs*GW* HOMO energies in our implementation are roughly 0.25 eV lower than the ev*GW*@PBE ones from Fediai *et al*. We observe that our qs*GW* HOMO energies are, on average, around 0.15 eV lower than the ev*GW*@PBE HOMO energies *without* CBS extrapolation. Furthermore, we observe, again on average, that the CBS extrapolation lowers their Homo energies by roughly 0.40 eV. Thus, the observed shift between the mean of our qs*GW* Homo energies and the mean of the reference ev*GW*@PBE Homo energies can be explained by partial cancellation of both effects.

As a reference to compare our qsGW-BSE excitation energies, we choose the QM9S dataset^25^. It provides TD-DFT excitation energies on the ωB97X-D/6-31G(d) level of theory for most QM9 molecules that additionally were reoptimized on the B3LYP-D3(BJ)/6-31G(d) level of theory. Figure 2 on the right shows the distribution of the respective lowest singlet-singlet qsGW-BSE excitation energies together with the respective lowest QM9S TD-DFT excitation energies. Both distributions have the same qualitative structure, with a sharp peak within the range 3 eV to 5 eV and a broader group of overlapping peaks in the range 5 eV to 9 eV. The difference between both distributions is that our qsGW-BSE excitation energies are shifted towards larger energies. To be precise, we observe that the qsGW-BSE excitation energies are, on average, 1.36 eV higher than the TD-DFT excitation energies. The analogous distribution for the lowest singlet-triplet qsGW-BSE excitation energies is shown in Fig. S1 in the Supporting Information. However, to the best of our knowledge, no comparable QM9-wide reference dataset is currently available for a quantitative comparison.

Overall, through comparison to similar work from the literature, we can confirm the reliability of our data since all presented data distributions demonstrate the absence of implausible outliers and are free of implausible distortions.

## Supplementary information

Supplementary Information

## Data Availability

All data generated by us is publicly available at Zenodo https://zenodo.org/records/17902233.

## Code availability

The AMS software package containing the ADF engine and the PLAMS toolkit is available from https://www.scm.com/downloads/ for a license fee. Template ADF input files used in this work can be found in the SI. Further code is not required to reproduce the data presented here.
