# Supplementary material for: qsGW quasiparticle and GW-BSE excitation energies of 133,885 molecules
Source: Sci Data. 2026 Mar 10;13:643. doi: 10.1038/s41597-026-07018-4 (PMC13103062; doi:10.1038/s41597-026-07018-4)
Supplement: Supplementary file 1 — Supplementary Information [file 41597_2026_7018_MOESM1_ESM.pdf]

Supporting Information

qsGW quasiparticle and GW-BSE excitation energies of 133 885 molecules

Dario Baum, Arno Förster, Lucas Visscher

Example ADF input file initial calculations

```
Task SinglePoint

system
  Atoms
            H       1.0151783459      -0.0657170576       0.0000000000
            O       0.0505387116       0.0505387116       0.0000000000
            H      -0.0657170576       1.0151783459       0.0000000000
  End
End

Engine adf
  basis
    core None
    type Corr/TZ3P
  End
  dependency
    bas 0.005
  End
  excitations
    bse True
    iterations 20
    lowest 5
  End
  gw
    nstates -1
    selfconsistency qsGW
  End
  numericalquality Good
  relativity
    level None
  End
  rihartreefock
    dependencythreshold 0.005
  End
  symmetry nosym
  xc
    libxc BHandHLYP
  End
EndEngine
```

Example ADF input file restart calculations

```
Task SinglePoint

system
  Atoms
            H       1.0151783459      -0.0657170576       0.0000000000
            O       0.0505387116       0.0505387116       0.0000000000
            H      -0.0657170576       1.0151783459       0.0000000000
  End
End

Engine adf
  basis
    core None
    type Corr/TZ3P
  End
  dependency
    bas 0.001
  End
  excitations
    bse True
    iterations 20
    lowest 5
  End
  gw
    nstates -1
    selfconsistency qsGW
  End
  numericalquality VeryGood
  relativity
    level None
  End
  rihartreefock
    dependencythreshold 0.001
  End
  symmetry nosym
  xc
    libxc BHandHLYP
  End
EndEngine
```

Notes on convergence of qsGW calculations

Difficulties in converging qsGW, mostly for small molecules without well-defined QP peaks for frontier orbitals, have been reported and discussed previously by different authors[1, 2]. In the absence of such cases, convergence is reliable and also depends on the frequencies at which the off-diagonal elements of the self-energy are evaluated (We refer to Refs. [3, 4, 5] for more details). In the implementation used here, we evaluate the off-diagonal elements of the self-energy at the Fermi energy, which helps convergence[3]. The settings we use here are sufficient to converge qsGW and the qsGW-BSE calculation to a precision of a few meV[6, 7].

Relative errors of singlet-singlet and singlet-triplet GW-BSE excitation energies

Bin	N_{samples}	E_{rel} [%]
$2\,\text{eV} \leq E_{\text{exc,SS}} < 4\,\text{eV}$	6	0.97
$4\,\text{eV} \leq E_{\text{exc,SS}} < 6\,\text{eV}$	80	1.07
$6\,\text{eV} \leq E_{\text{exc,SS}} < 8\,\text{eV}$	315	1.95
$8\,\text{eV} \leq E_{\text{exc,SS}} < 10\,\text{eV}$	99	3.10

Table S1: Mean relative errors of different ranges of GW-BSE singlet-singlet excitation energies based on the TZ3P and QZ6P basis sets. The QZ6P energies are taken as references for the calculation of the relative errors.

Bin	N_{samples}	E_{rel} [%]
$2\,\text{eV} \leq E_{\text{exc,ST}} < 4\,\text{eV}$	44	1.04
$4\,\text{eV} \leq E_{\text{exc,ST}} < 6\,\text{eV}$	144	0.86
$6\,\text{eV} \leq E_{\text{exc,ST}} < 8\,\text{eV}$	259	1.66
$8\,\text{eV} \leq E_{\text{exc,ST}} < 10\,\text{eV}$	41	3.02

Table S2: Mean relative errors of different ranges of GW-BSE singlet-triplet excitation energies based on the TZ3P and QZ6P basis sets. The QZ6P energies are taken as references for the calculation of the relative errors.

Distribution of GW-BSE singlet-triplet excitation energies

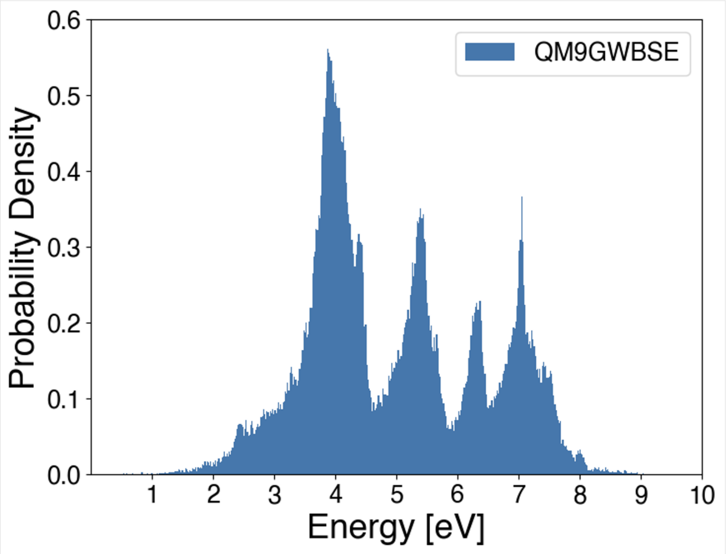

Figure S1: Distribution of lowest GW-BSE singlet-triplet excitation energies for each molecule from the QM9GWBSE dataset.

References

[1] Antoine Marie and Pierre-François Loos. A similarity renormalization group approach to green's function methods. *Journal of Chemical Theory and Computation*, 19(13):3943–3957, 2023.

[2] Oliver J Backhouse, Marcus K Allen, Charles JC Scott, and George H Booth. Self-consistent gw via conservation of spectral moments. *Journal of Chemical Theory and Computation*, 21(18):8963–8981, 2025.

[3] Arno Förster and Lucas Visscher. Low-order scaling quasiparticle self-consistent gw for molecules. *Frontiers in Chemistry*, 9:736591, 2021.

[4] Jincheng Lei and Tianyu Zhu. Gaussian-based quasiparticle self-consistent gw for periodic systems. *The Journal of Chemical Physics*, 157(21), 2022.

[5] Gaurav Harsha, Vibin Abraham, Ming Wen, and Dominika Zgid. Quasiparticle and fully self-consistent gw methods: an unbiased analysis using gaussian orbitals. *Physical Review B*, 110(23):235146, 2024.

[6] Jelena Belić, Arno Förster, Jan Paul Menzel, Francesco Buda, and Lucas Visscher. Automated assessment of redox potentials for dyes in dye-sensitized photoelectrochemical cells. *Physical Chemistry Chemical Physics*, 24(1):197–210, 2022.

[7] Arno Förster and Lucas Visscher. Quasiparticle self-consistent gw-bethe–salpeter equation calculations for large chromophoric systems. *Journal of chemical theory and computation*, 18(11):6779–6793, 2022.
